# *BRCA2* Variants and cardiovascular disease in a multi-ethnic study

**DOI:** 10.1186/1471-2350-13-56

**Published:** 2012-07-18

**Authors:** Kevin Zbuk, Changchun Xie, Robin Young, Mahyar Heydarpour, Guillaume Pare, A Darlene Davis, Ruby Miller, Matthew B Lanktree, Danish Saleheen, John Danesh, Salim Yusuf, James C Engert, Robert A Hegele, Sonia S Anand

**Affiliations:** 1 Population Health Research Institute, Hamilton Health Sciences, Hamilton, ON, Canada; 2 Department of Public Health and Primary Care, Cambridge University, Cambridge, UK; 3 Six Nations Health Services, Ohsweken, ON, Canada; 4 Schulich School of Medicine and Dentistry, University of Western Ontario, London, ON, Canada; 5 Departments of Medicine and Human Genetics, McGill University, Montreal, QC, Canada; 6 Department of Oncology, McMaster University, Hamilton, ON, Canada; 7 Departments of Medicine and Epidemiology, McMaster University, Hamilton, ON, Canada; 8 Department of Pathology and Molecular Medicine, McMaster University, Hamilton, ON, Canada; 9 Population Health Research Institute, McMaster University Hamilton General Hospital Campus, DB-CVSRI, 237 Barton Street East, Rm. C3102, Hamilton, ON, L8L 2X2, Canada; 10 Center for Non-Communicable Diseases, Karachi, Pakistan; 11 Department of Biostatistics and Epidemiology and Department of Medicine, University of Pennsylvania, Karachi, Pakistan

## Abstract

**Background:**

Germline mutations of *BRCA1/2* are associated with hereditary breast and ovarian cancer. Recent data suggests excess mortality in mutation carriers beyond that conferred by neoplasia, and recent *in vivo* and *in vitro* studies suggest a modulatory role for BRCA proteins in endothelial and cardiomyocyte function. We therefore tested the association of *BRCA2* variants with clinical cardiovascular disease (CVD).

**Methods:**

Using data from 1,170 individuals included in two multi-ethnic population-based studies (SHARE and SHARE-AP), the association between *BRCA2* variants and CVD was evaluated. 15 SNPs in *BRCA2* with minor allele frequencies *(MAF) > 0.01* had been previously genotyped using the cardiovascular gene-centric 50 k SNP array. 115 individuals (9.8%) reported a CVD event, defined as myocardial infarction (MI), angina, silent MI, stroke, and angioplasty or coronary artery bypass surgery. Analyses were adjusted for age and sex. The SNPs rs11571836 and rs1799943 were subsequently genotyped using the MassARRAY platform in 1,045 cases of incident MI and 1,135 controls from the South Asian subset of an international case-control study of acute MI (INTERHEART), and rs11571836 was imputed in 4,686 cases and 4500 controls from the Pakistan Risk of Myocardial Infarction Study (PROMIS).

**Results:**

Two *BRCA2* SNPs, rs11571836 and rs1799943, both located in untranslated regions, were associated with lower risk of CVD (OR 0.47 p = 0.01 and OR 0.56 p = 0.03 respectively) in the SHARE studies. Analysis by specific ethnicities demonstrated an association with CVD for both SNPs in Aboriginal People, and for rs11571836 only in South Asians. No association was observed in the European and Chinese subgroups. A non-significant trend towards an association between rs11571836 and lower risk of MI was observed in South Asians from INTERHEART [OR = 0.87 (95% CI: 0.75-1.01) p = 0.068], but was not evident in PROMIS [OR = 0.96 (95% CI: 0.90-1.03) p = 0.230]. Meta-analysis of both case-control studies resulted in a combined OR of 0.94 (95% CI: 0.89-1.004, p = 0.06).

**Conclusions:**

Although there was an association between two SNPs in *BRCA2* and CVD in a multi-ethnic population, these results were not replicated in two South Asian case-control studies of incident MI. Future studies exploring the association between *BRCA* variants and cardiovascular disorders are needed to clarify the role, if any, for *BRCA* variants in CVD pathogenesis.

## Background

Germline mutations of the genes *BRCA1* (breast cancer 1, early onset) and *BRCA2* (breast cancer 2, early onset) are the most common cause of hereditary breast and ovarian cancer. Autosomal dominant mutations in *BRCA1/2* are associated with a lifetime risk of breast and ovarian cancer of up to 85% and 45% respectively [1]. In addition, mutation carriers are at increased risk of several additional cancers including male breast cancer, pancreatic adenocarcinoma, prostate cancer, and melanoma [2]. A recent publication has suggested *BRCA* mutation carriers have an excess risk of mortality compared to non-carriers beyond that explained by the development or treatment of malignancy [3]. Mai *et al.* utilized a kin-cohort analysis to estimate the effect of *BRCA*1/2 mutations on mortality. When deaths related to cancer diagnosis were excluded, the difference in life expectancy was 5.7 years lower for women and 3.7 years for men with mutations compared to age matched non-mutation carriers. The etiology of this excess mortality is currently unknown. However, BRCA1 and BRCA2 are members of a complex network of proteins involved in DNA repair and genomic stability [4], suggesting their role could extend beyond regulation of neoplasia.

Recently, a cardiomyocyte specific *BRCA1* knockout mouse was developed, which showed increased susceptibility to myocardial ischemia and genotoxic agents (doxorubicin), suggesting BRCA dysfunction might play a role in cardiac damage during myocardial infarction and genotoxin induced cardiotoxicity [5]. In a separate study, cardiomyocyte specific *BRCA2* knockout mice exhibited similar susceptibility to genotoxic agents [6]. There was an association between increased double stranded DNA breaks and cardiac damage in both reports irrespective of the precipitating cause, alluding to the possibility of similarities in pathogenesis [5,6]. The idea of overlapping functions for BRCA1 and BRCA2 is further supported by evidence that the two proteins co-localize in the nucleus and play crucial roles in DNA repair [4]. In addition, the cancer phenotypes expressed by germline mutations in both genes are similar [7].

*BRCA* mutations that are associated with a markedly increased risk of cancer (most often resulting in protein truncation) are extremely rare in the general population [8]. There are however, several hundred single nucleotide polymorphisms (SNPs) in *BRCA1/2,* many of which are common [9]. We hypothesized that common polymorphisms in *BRCA* genes may be associated with cardiovascular disease (CVD), another complex trait, with molecular evidence (see above) suggesting a role for the *BRCA* genes.

To test this hypothesis, we assessed the association between SNPs in *BRCA* and CVD in individuals from the multi-ethnic SHARE and SHARE-AP studies [10,11]. These cross-sectional, randomly sampled, population-based studies explored differences in CVD risk factors and prevalence in South Asian, European, Chinese, and Aboriginal ethnic groups residing in Canada. The SHARE studies demonstrated that Aboriginal people and South Asians had the highest prevalence of CVD amongst the four ethnic groups [10,11].

## Methods

### Overall approach

We analyzed SHARE and SHARE-AP data that was available from the prior genotyping of 50,000 SNPs from candidate genes and pathways for cardiovascular, inflammatory and metabolic phenotypes in these study populations [12-14]. The Illumina HumanCVD beadchip array includes twenty-one SNPs in *BRCA2* with *MAF >0.01* but does not include any SNPs in *BRCA1.*

Due to the availability of DNA samples from South Asians, we examined the association between rs11571836 and rs1799943 and incident MI in South Asians from the INTERHEART. INTERHEART is a large, global, standardized case-control study of incident MI from 52 countries [15]. We next became aware of genome wide genotyping data from the PROMIS study, a similar case-control study of incident MI amongst South Asians residing in urban regions of Pakistan [16]. Since our analysis of INTERHEART showed a trend towards an association between MI and rs11571836, but no association between MI and rs1799943, we examined the association of rs11571836 and MI using imputation in the PROMIS analysis.

### Informed consent and REB approval

All studies were approved by appropriate research ethics boards. In addition, all participants provided informed consent, including consent for the collection and processing of genetic material in accordance with TriCouncil guidelines.

### SHARE studies

The methodology and results of the SHARE studies are described in detail elsewhere [10,11]. At the time of enrolment, no participant had a diagnosis of cancer. Canadians were classified as South Asian if their ancestors originated from India, Pakistan, Sri Lanka, or Bangladesh; Chinese if their ancestors originated from China, Taiwan, or Hong Kong; and European if their ancestors originated from Europe. Aboriginal people were identified as such if they were members of the Six Nations Band. CVD was defined as prevalent cases of myocardial infarction, angina, coronary angioplasty or CABG, silent myocardial infarction defined by major Q waves on ECG, and stroke as previously described. The ascertainment of CVD events was from self-administered questionnaires, medical records, and electrocardiograms [17].

Genotyping of the SHARE and SHARE-AP samples on the Illumina HumanCVD beadchip had been previously carried out, utilizing standard protocols at two locations, as described in detail elsewhere [13,14]. The three ethnic groups in SHARE were genotyped at The Centre for Applied Genomics (Hospital for Sick Children, Toronto, Ontario; www.tcag.ca), and the Aboriginal people from SHARE-AP were genotyped at the McGill University and Genome Quebec Innovation Centre (Montreal, Quebec;www.genomequebec.com).

### Replication studies (INTERHEART and PROMIS)

The methodology and results of the INTERHEART and PROMIS studies are described in detail elsewhere [15,16]. For both studies, individuals presenting to hospitals with acute MI defined cases, while controls were generally patients from the same hospital with no history of cardiovascular disease. The identification of South Asian ethnicity in INTERHEART was based on self-reported ethnicity. However, individuals with self-reported ethnicity that differed from genetically inferred ancestry were excluded from the analysis as previously described [18]. Genotyping of rs11571836 in 1,143 cases and 1,218 controls of South Asian ethnicity from INTERHEART was performed at the McGill University and Genome Quebec Innovation Centre (Montreal, Quebec;www.genomequebec.com) using the MassARRAY platform as previously described [19].

Participants of PROMIS were recruited from hospitals of four large urban centres in Pakistan. South Asians were defined as those with ancestry from the Indian subcontinent. As part of PROMIS, 4,686 cases of incident MI and 4,500 controls had been previously genotyped at the Center for Non-Communicable Diseases (Karachi, Pakistan) using the Illumina 660 genome wide association platform as described elsewhere [20]. This array has SNPs spaced at an average of approximately 4 kb intervals. The genotype of rs11571836 was imputed in cases and controls using the software IMPUTE [21]. The Hapmap Phase 2 population (270 individuals) combined with the South Asian samples from Phase 3 (90 individuals) served as the reference samples for the imputation (hapmap.ncbi.nlm.nih.gov).

### Statistical analysis

Analyses were performed using SAS (version 8.2; SAS Institute, Cary, NC) and PLINK [22]. Linkage disequilibrium (LD) maps and *r*^*2*^ values were produced using Haploview 4.0 [23]. In SHARE and SHARE-AP *BRCA2* SNPs were tested for their association with prevalent CVD, in each ethnicity, using logistic regression analysis assuming an additive model. Analyses were adjusted for age and sex. The association results for each ethnicity were thereaftercombined utilizing a fixed-effect meta-analysis, adjusted for multiple comparison. The association between incident MI and rs11571836 and rs1799943 was tested in South Asians from the INTERHEART study using logistic regression with an additive model, adjusted for age and sex. Association testing of imputed genotypes with MI in the PROMIS study was performed using SNPTEST [23,24] utilizing a logistic regression model adjusted for age and sex. A fixed effects meta-analysis combining the association results for rs11571836 and CVD (for the SHARE studies) or MI (for INTERHEART AND PROMIS) from all three studies was performed using PLINK.

### Quality control

Of the 21 *BRCA2* SNPs present on the HumanCVD beadchip with MAF greater than 0.01 six were excluded as call rates were less than 95% in one or more ethnicities. For the remaining 15 SNPs call rates were in excess of 97% in all ethnicities. In addition, 15 individuals were excluded because of genotype call rates less than 95% for the 15 SNPs. There was no significant deviation from Hardy Weinberg equilibrium as tested within each ethnic group (all p values >0.1). Genotyping of rs11571836 in the INTERHEART samples resulted in a call rate of 98%. The quality of imputation of rs11571836 from PROMIS, as measured by the SNPtest info score was 0.978. No samples were excluded due to poor imputation confidence.

## Results

### SHARE and SHARE-AP

Of 1,178 individuals with complete genotyping, 115 individuals had at least one CVD outcome (9.8%). Tables 1 and 2 show CVD associations by ethnicity for rs11571836 and rs1799943. CVD was more prevalent in the Aboriginal and South Asian groups compared with the European and Chinese groups. In the case of rs11571836 (Table 1) the association was statistically significant for the Aboriginal and South Asian sub-groups while for rs1799943 (Table 2) the association was statistically significant only in the Aboriginal group. Both SNPs were associated with CVD in meta-analyses that the combined association results for all four ethnicities. For rs11571836 the combined OR was 0.56 (p value 0.009) while for rs1799943 the combined OR was 0.60 (p value 0.01). The I^2^ test for heterogeneity was 0% for both meta-analyses. There was no association between CVD and any of the other *BRCA2* SNPs examined (Additional file 1: Table S1).

**Table 1:** Association test results for rs11571836 and CVD (SHARE + AP)

**Ethnicity**	**Individuals with at least one CVD event (% of total)**	**Individuals with no CVD events (n)**	MAF (G allele)*	**OR (95% CI)**	**P**
All combined^+^	115 (10%)	1063	range: 0.15-.038	0.56	0.009
Aboriginal	55 (19%)	235	0.15	0.42 (0.19-0.91)	0.017
South Asian	32 (10%)	291	0.23	0.43 (0.19-0.99)	0.021
European	20 (8%)	245	0.20	0.37 (0.08-1.63)	0.19
Chinese	8 (3%)	292	0.38	0.99 (0.35-2.9)	0.99

^+^ meta-analysis (fixed-effect model) adjusted for multiple comparisons.

*MAF calculated from cases and controls combined for each ethnicity.

**Table 2:** Association test results for rs1799943 and CVD (SHARE + AP)

**Ethnicity**	**Individuals with at least one CVD event (% of total)**	**Individuals with no CVD events (n)**	MAF (A allele) *	**OR (95% CI)**	**P**
All combined^+^	115 (10%)	1063	range: 0.18-0.37	0.6	0.01
Aboriginal	55 (19%)	235	0.18	0.40 (0.19-0.83)	0.007
South Asian	32 (10%)	291	0.32	0.58 (0.29-1.13)	0.10
European	20 (8%)	245	0.25	0.79 (0.35-1.78)	0.55
Chinese	8 (3%)	292	0.37	1.00 (0.35-2.86)	0.99

^+^ meta-analysis (fixed-effect model) adjusted for multiple comparisons.

*MAF calculated from cases and controls combined for each ethnicity.

**Figure 1:**
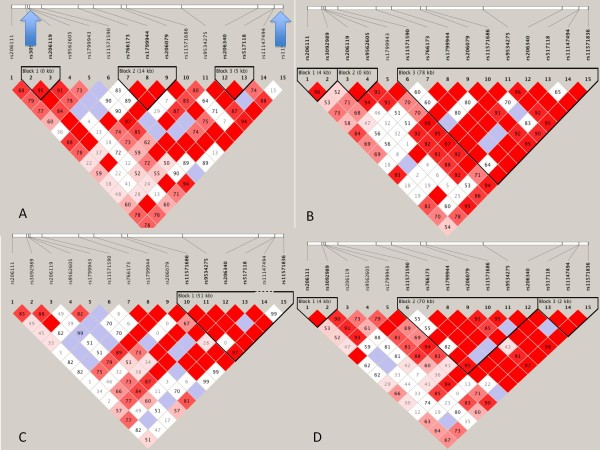
**Linkage disequilibrium maps of*BRCA*region for the 4 ethnic groups.A**) Aboriginal **B**) Chinese **C**) European **D**) South Asian. Arrowheads in panel **A** indicate position of rs11571836 (right arrow) and rs1799943 (left arrow).

Figure 1 shows the LD patterns by ethnicity for SNPs in *BRCA2* genotyped in this study. There are significant differences in LD distribution, as defined by LD blocks, between the four ethnic groups. Additionally, there is more LD in this region in the Chinese, South Asian and Aboriginal groups compared to the European group. However, there was no significant LD between rs1799943 and rs11571836 (r^2^ <0.2) in any ethnicity. The SNP rs11571836 is located in the 3’ UTR of *BRCA2*, while rs1799943 is in the 5’UTR.

### Replication studies (INTERHEART AND PROMIS)

**Figure 2:**
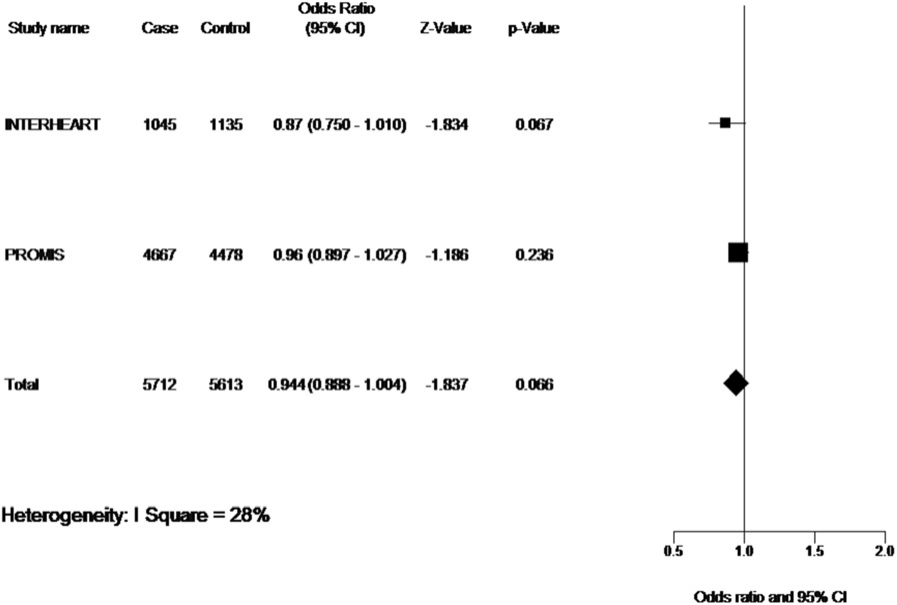
Meta-analysis of the association between rs11571836 and MI from INTERHEART and PROMIS (fixed effects model).

Figure 2 illustrates the results of association analyses between rs11571836 and incident MI in the South Asian INTERHEART and PROMIS studies, including meta-analysis combining the two studies. There was a trend towards an association between rs11571836 and MI in the INTERHEART analysis [OR = 0.87 (95% CI: 0.75-1.01], which did not reach statistical significance (p = 0.068). In contrast, there was no association between MI and rs1799943, p = 0.3 [OR-0.93 (95% CI 0.821-1.062)]. There was no significant association between rs11571836 and incident MI in the PROMIS association study [OR = 0.96 (95% CI: 0.90-1.03) p = 0.23].

Finally, meta-analysis of the replication studies (PROMIS and INTERHEART) for rs111571836 resulted in a combined OR of 0.94 (95% CI: 0.89-1.004, p = 0.066). The I^2^ test for heterogeneity was 28% (low to moderate). We also performed a meta-analysis utilizing the CVD association results for the South Asian subgroup of SHARE, combined with the association results for acute MI from the two replication studies. Although the resulting odds ratio (OR = 0.9) and p-value (p = 0.05) were marginally lower, the level of heterogeneity was much higher (I^2^ = 68%, Chi-squared heterogeneity statistic >3 with 2 degrees of freedom). We therefore did not include the SHARE results in the final meta-analysis and resulting forest plot (Figure 2).

## Discussion

To our knowledge, this report is the first to suggest a possible association between variants in *BRCA1/2* and CVD in human populations. However this observation requires confirmation, given that the effect size appears to be small, and our meta-analysis did not reach statistical significance. It is possible that the association we observed in the SHARE studies was due to chance alone, and is not a true association.

Very little has been published with respect to shared genetic predisposition to cancer and CVD. However, mechanistically, several common pathways are emerging. Most prominent in recent publications is the endothelin axis, with reports linking endothelin-1 to prostate cancer tumorigenesis [25]. Additionally, animal models demonstrate upregulation of DNA repair mechanisms in heart failure induced by ischemic damage [26]. As an example, topoisomerase-II-alpha, a protein involved in various aspects of DNA repair, is overexpressed in unstable plaque from human carotid atheromas [27]. Given its integral role in genomic stability and DNA repair, a similar role in the pathogenesis of coronary atherosclerosis, plaque rupture, and response to ischemic damage may exist for *BRCA1/2*. Finally, several SNPs associated with metabolic diseases, identified through genome wide association studies, have roles in cancer and chronic disease predisposition. For example, variants in *TCF7L2* predispose to both colon cancer and type 2 diabetes while variants in *HNF1B* predisposes to type 2 diabetes and prostate cancer [28].

Our study suggested a possible association between rs11571836 and rs1799943 and CVD in a multi-ethnic population. Analysis by ethnicity confirmed a statistically significant association with Aboriginal and South Asian ethnicities for rs11571836, while for rs1799943 the association was statistically significant only in the Aboriginal group. It is worth noting however, that with the exception of the Chinese group, all odds ratios trended in the same direction (ie. below 1) for both SNPs. This supports the possible explanation that the current analysis was underpowered to detect an association by individual ethnicity. In fact, only 17% and 6.9% of individuals with CVD in the study were European or Chinese respectively. Alternatively, lack of association between these SNPs and CVD in specific ethnicities may represent differences in LD in the region encompassing *BRCA2,* as there are significant differences in LD structure in this region among ethnicities (Figure 1)*. In silico* analysis of these two SNPs suggests both may modulate *BRCA2* transcript levels [29], but there have been no published functional studies to date. Similarly, neither SNP has been definitively associated with breast cancer susceptibility. Thus, it is quite possible that rs11571836 and rs1799943 are not directly associated with CVD, but instead are in LD with other functional variants associated with CVD. Finally, there may be ethnic-specific differences in the pathogenesis of CVD such that *BRCA* variation is only relevant to CVD in certain ethnicities, although this seems unlikely given the consistency of other risk factors for CVD across ethnicities [15].

We were unable to demonstrate a statistically significant association between rs11571836 and incident MI in two large case controls studies of incident MI in South Asians, although a trend towards an association was present in INTERHEART. It is possible in INTERHEART that a larger sample size may have resulted in a statistically significant association, as the current sample size was underpowered (44% power) to detect a 15% risk reduction in MI risk for allele frequencies of 20%. A sample size of approximately 2600 cases and a similar number of controls is required to reach 80% power. Based on sample size, the PROMIS analysis was adequately powered, however, it is possible that utilizing imputation to infer the genotype of rs11571836 may have resulted in divergent results from what would have been observed had the SNP been directly genotyped. While the literature suggests good concordance between imputation and direct genotyping [30], it is possible that the HapMap reference samples were not adequate for this region. Alternatively, subtle differences in LD patterns between South Asian populations could explain the lack of a reproducible association between rs11571836 and acute MI in the SHARE, INTERHEART and PROMIS studies. Conversely, the lack of replication would be anticipated if the association between *BRCA2* variants and CVD demonstrated in the SHARE studies occurred by chance. One means of further clarifying this possibility is to examine the association between *BRCA2* variants and CAD in published GWAS studies. There was no association between either rs11571836 or rs1799943 and CAD in the Wellcome Trust Case Control Consortium (WTCCC) GWAS study [31]. However, the WTCCC study was composed exclusively of White Europeans, unlike our multi-ethnic or South Asian study populations. Unfortunately, lack of publically available data from addition multi-ethnic or South Asian GWAS studies limited our ability “look-up” these SNPs directly in additional studies.

Alternatively, variants in *BRCA2* may be more relevant to the pathogenesis of other components of the composite CVD outcome utilized in SHARE (for example ischemic stroke), rather than acute MI. One significant limitation of utilizing the composite CVD outcome in genetic association studies is the heterogeneous pathogenesis of the outcomes studied. In the SHARE studies, the low number of events precluded an assessment of individual components of the composite CVD outcome. Testing the association between *BRCA* variants and measures of CVD other than MI, ideally from multiple ethnicities, could confirm or refute this hypothesis. In addition, experimental data has thus far only demonstrated a role for *BRCA1* in the modulation of cardiomyocyte function following ischemic damage. Due to resource constraints, our analysis was limited to SNPs from *BRCA2* that had been previously genotyped, however, investigating the role of SNPs in *BRCA1* would be desirable. Finally, data from the murine knock-out models described earlier allude to the possibility that BRCA1/2 may play a greater role in modulating the severity of cardiomyocyte damage following an insult, rather than being directly implicated in initiation of cardiac injury. Therefore, testing the association between variants in the *BRCA* genes and the severity of a CVD outcome might prove more useful.

## Conclusions

Our results suggested a possible association between *BRCA2* and CVD in the multi-ethnic SHARE study, however we were unable to replicate this association in two large case-control studies of acute MI in South Asians. Future studies exploring the association between *BRCA1/2* variants and CVD outcomes, may help clarify the role, if any, of *BRCA* variants in CVD pathogenesis.

## Competing interests

The authors declare no competing interests.

## Authors' contributions

KZ was involved in conception of the study, data analysis, and drafting the manuscript. CX performed statistical analysis. RY was involved in genotyping and association analyses. MH performed statistical analysis. RM and ADD were involved in data acquisition and study co-ordination. GP, SYand JE were involved in study design, data acquisition, and aided in the drafting and revision of the manuscript. RAH, MBL, DS, and JD were involved in data acquisition, genotyping, and revisions of the manuscript. SSA was involved in conception of the study, data acquisition, statistical analysis, and drafting and revising the manuscript. All authors read and approved the final manuscript.

## Supplementary Material

Additional file 1: **Table S1.** Association Test Results for *BRCA2* SNPs and CVD (SHARE+AP). Click here for file
