# Supplementary material for: BRCA2 Variants and cardiovascular disease in a multi-ethnic study
Source: BMC Med Genet. 2012 Jul 18;13:56. doi: 10.1186/1471-2350-13-56 (PMC3464815; doi:10.1186/1471-2350-13-56)
Supplement: Additional file 1: — Table S1. Association Test Results for BRCA2 SNPs and CVD (SHARE+AP). [file 1471-2350-13-56-S1.doc]

Supplementary Table 1.

Association Test Results for *BRCA2* SNPs and CVD (SHARE+AP)

* Adjusted for age, sex and ethnicity
